# Effect of oxygenated ferrocene derivatives on soot formation and nanoparticle emissions in *n*-heptane diffusion flames

**DOI:** 10.1039/d5ra09720g

**Published:** 2026-03-16

**Authors:** Anoop C. V., Raja Mitra, Thaseem Thajudeen, Anirudha Ambekar

**Affiliations:** a School of Mechanical Sciences, Indian Institute of Technology Goa India anirudha@iitgoa.ac.in; b School of Chemical and Materials Sciences, Indian Institute of Technology Goa India; c Center of Excellence in Sustainable Energy, Indian Institute of Technology Goa India

## Abstract

This study reports an experimental investigation of soot formation in laminar diffusion flames of *n*-heptane doped with organometallic additives, including ferrocene, ferrocene methanol, and ferrocene carboxaldehyde, at concentrations of 100 and 500 ppm. Measurements of flame temperature, flame Soot Volume Fraction (SVF), and aerosol concentration in the flame plume were obtained. The addition of organometallics lowered the SVF compared to pure *n*-heptane. The oxygenated ferrocene derivatives were found to be significantly more effective at suppressing soot compared to pure ferrocene. At 100 ppm, ferrocene methanol and ferrocene carboxaldehyde reduced peak SVF by 24% and 22%, compared to only 8% for ferrocene. Increasing the concentration to 500 ppm provided only a marginal additional improvement. However, the measurements of aerosol concentration in the flame plume showed ultrafine particulate emissions for additive-doped flames. These emissions were attributed to iron-containing nanoparticles. Total Number Concentration (TNC) of particulate emissions was comparable for ferrocene and ferrocene carboxaldehyde, while ferrocene methanol showed a marginally lower value. The study demonstrates that organometallic additives reduce soot concentration within the flame while increasing nanoparticle emissions. This highlights a trade-off between soot suppression and nanoparticle emissions in the context of the selection and use of such additives. Furthermore, the study highlights the advantage of combined diagnostics, including aerosol measurement, over simple in-flame soot measurement to assess the overall emissions.

## Abstract 2

The in-flame soot suppression, emission of ultrafine particles, and reduction in flame temperature are features common to all additives studied here.

## Graphical abstract

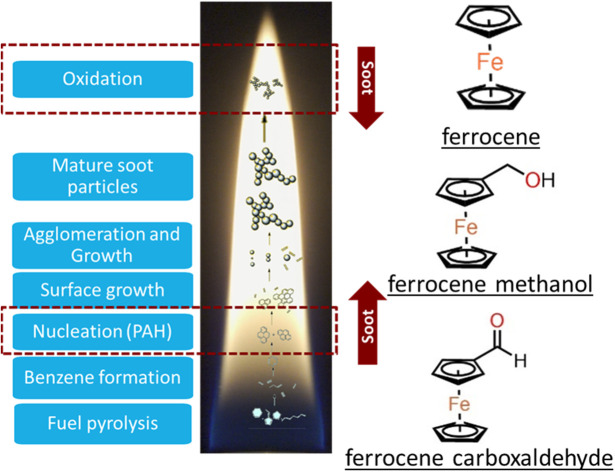

## 1. Introduction

The combustion of hydrocarbon fuels, including both conventional and renewable biofuels, is known to produce soot emissions. The reduction of these soot emissions is crucial in mitigating issues such as poor air quality, health problems, and climate change.^1,2^ Typical steps in soot formation are fuel pyrolysis, formation of soot precursors or Polycyclic Aromatic Hydrocarbons (PAHs), particle nucleation, surface growth, coagulation, carbonization, and the final step of soot oxidation, where the soot is partially or fully burned. The reduction of soot emissions can be achieved through appropriate disruption of this process. Specifically, soot mitigation is typically pursued through the use of fuel-borne additives, the formulation of cleaner fuel blends, and novel combustion techniques.

Among the numerous fuel-borne additives, the use of ferrocene ((C_5_H_5_)_2_Fe) has been widely investigated in the past. In particular, the effect of ferrocene on the soot emissions from various diffusion flames has been reported in the literature. Early research has shown that ferrocene vapor generates iron-based species that serve as nucleation sites for PAH growth and soot inception, while in the soot oxidation zone, iron-based species accelerate soot burnout and lower the thermal stability of the soot.^3–6^ A range of fundamental studies confirms that the addition of ferrocene often leads to an increased particle number concentration in the early stage while simultaneously contributing to soot destruction later. These opposing tendencies result in altered soot mass, particle number, particle size, and microstructure. Thus, the action of ferrocene is characterized by the modification of soot emissions rather than an outright elimination. One effect attributed to this behaviour is reduction in large graphitic aggregates but increased ultrafine iron-bearing particle emissions.

Kasper *et al.*^5^ have reported iron oxide particles outside the flame plume, confirming nanoparticle emissions associated with the additive. The experimental investigation reported the emission of iron oxide nanoparticles in methane–air diffusion flames doped with ferrocene, where the presence of iron oxide was inferred from its lower photoelectric yield compared to carbonaceous soot particles, measured using a femto-amperemeter. Similarly, Wallis *et al.*^7^ gave direct evidence through high-resolution *Z*-contrast imaging and electron energy-loss studies of the presence iron contained particles in carbonaceous matrix within hydrocarbon flames and their contribution to catalytic soot oxidation. Several other studies^4,8–10^ have also indicated presence of iron oxide particles which contribute to soot oxidation enhancement.

More recently, Hu *et al.*^11,12^ have studied the effect of ferrocene on flame temperature, soot formation, and PAH growth in a propane oxygen diffusion flame and reported similar observations. Studies involving practical applications of ferrocene along with ferrocene derivatives such as alkylferrocenes and dimethylferrocenylcarbinol have shown them to be effective as octane improvers.^13,14^ However, due to issues created by iron particle emissions, the maximum concentration in engine applications was limited to 38 ppm.^14^

The separate category of oxygenating additives is also known to provide a significant improvement in soot burnout near the flame terminus through changes in local stoichiometry and increasing OH concentration. Although the underlying mechanism is different from ferrocene, similar overall effects have been observed through oxygenation of the flame zone by adding oxygen-containing functional groups.^15–18^ Ying *et al.*,^15^ conducted study on the impact of soot oxidative property with different long chain alcohol additives on ethylene inverse diffusion flames, by conducting soot morphological studies and citing that the oxidative property reduces as the increment in carbon chain length of alcohol additive. The review done on oxygenated biofuel additives by Xu *et al.*^16^ also substantiates the enhancement in soot oxidative property of oxygen containing functional groups like –OH and C=O. Thus, the oxygenated functional groups in fuel additives have an enhancement effect on soot oxidative properties. Such additives are also reviewed recently in the context of their application to engines.^19–22^

A combination of the effect produced by ferrocene addition and the oxygenation could potentially lead to further improvements in terms of emissions and performance. However, studies exploring this possibility are sparse such as the investigation by Rustamov *et al.*,^13^ which demonstrated that a three-part mixture of gasoline, ferrocene, and di-isopropyl ether as the oxygenated additive increases the octane number of gasoline. At the same time, no studies reporting the performance of ferrocene functionalized with oxygenated functional groups could be found. Therefore, the efficacy of ferrocene-based additives with oxygen-containing functional groups may be explored further to ascertain their performance.

In the present study, ferrocene methanol and ferrocene carboxaldehyde were selected as representative oxygenated ferrocene derivatives containing alcohol (–OH) and aldehyde (–CHO) functional groups, respectively. These additives represent two common oxygen-containing groups that may significantly influence decomposition pathways, radical formation, and soot evolution in hydrocarbon flames. A review of the existing literature reveals a clear opportunity of systematic studies addressing the effects of ferrocene methanol and ferrocene carboxaldehyde on hydrocarbon diffusion flames. The present work therefore experimentally investigates the impact of ferrocene, ferrocene methanol, and ferrocene carboxaldehyde on the soot formation characteristics of *n*-heptane using a liquid strand burner.

The study aims to examine the effect of oxygenated functional groups on basic ferrocene-modified soot formation and nanoparticle generation. Flame temperature, soot volume fraction, and particulate emissions are quantified with pyrometry, Laser-Induced Incandescence (LII), and Scanning Mobility Particle Sizer (SMPS), respectively. Particular emphasis is placed on the trade-off between soot suppression and emissions of nanoparticles.

## 2. Experimental method

The experiment used *n*-heptane with 99% purity as a standard surrogate for gasoline,^23,24^ mixed with ferrocene-based additives, and combusted in the form of a liquid strand. The additives were mixed at 0.01% (100 ppm) and 0.05% (500 ppm) concentrations. The high solubility of ferrocene and ferrocene derivatives such as ferrocene with hydroxyl substitution groups in non-polar hydrocarbon solvents is well documented.^25^ The dissolution of the additives was considered sufficient through simple stirring. Table 1 shows the detailed chemical structures of the compounds used in this study, along with the nomenclature adapted for the blends.

**Table 1:** The organometallic additives used for the analysis

Compound	Formula	Structure	Blend nomenclature
*n*-Heptane	C_7_H_16_	—	—
Ferrocene	C_10_H_10_Fe	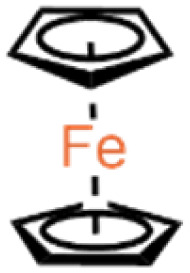	100 ppm blend – *n*-heptane-F100
			500 ppm blend – *n*-heptane-F500
Ferrocene methanol	C_10_H_9_CH_2_OHFe	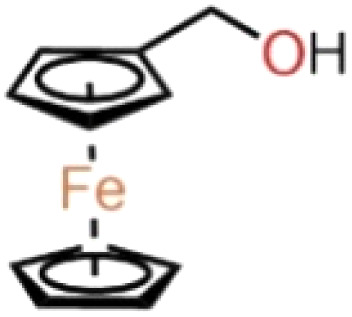	100 ppm blend – *n*-heptane-FM100
			500 ppm blend – *n*-heptane-FM500
Ferrocene carboxaldehyde	C_10_H_9_CHOFe	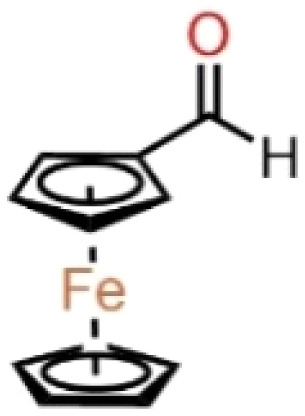	100 ppm blend – *n*-heptane-FC100
			500 ppm blend – *n*-heptane-FC500

**Fig. 1:**
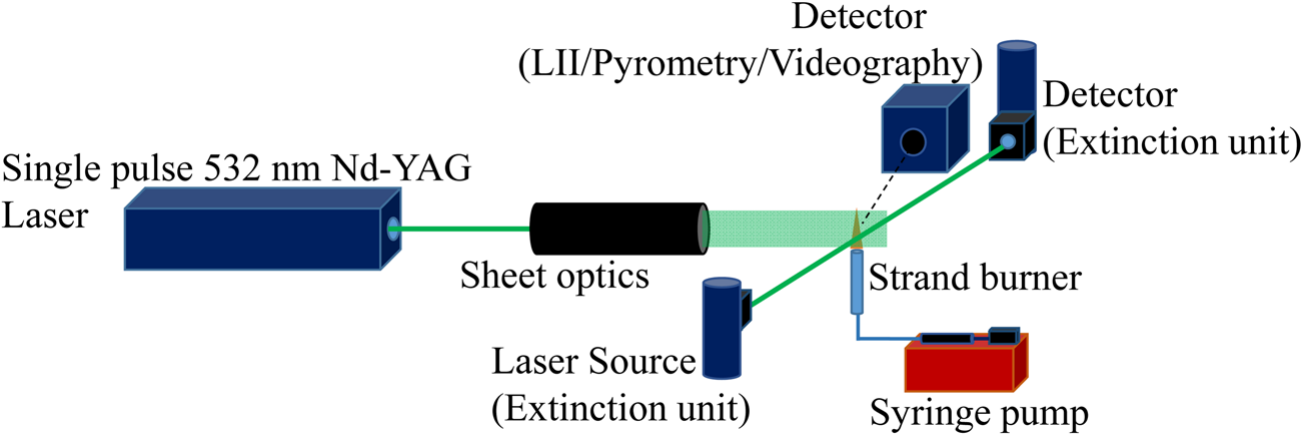
Experimental arrangement for the strand burner.

Fig. 1 shows the schematic of the experimental setup. The liquid strand was made using a glass tube with an internal diameter of 10 mm and height of 75 mm. The tube was modified to connect a silicone tube near the bottom to enable a continuous supply of the liquid fuel using a syringe pump. Ignition was achieved using a pilot flame briefly brought into contact with the tip of the fully filled tube. A Sony HDR AX 700 4K video camera captured the fuel level variation in the strand as well as the flame height variation.

The LaVision two-color pyrometer with Imager M-lite 2M colour camera was used for flame temperature measurement. The pyrometer was calibrated against a standard tungsten lamp up to a temperature of 2694 K. The pyrometer images were captured at 100 FPS, and each experiment was repeated thrice to ensure repeatability of the measurement. A LaVision LII system was used to measure of flame Soot Volume Fraction (SVF). Calibration data from a line-of-sight extinction measurement were utilized to obtain the quantitative soot volume fraction for the flame. The LII laser was set to 430 mJ at 10 Hz, and a laser filter of 532 nm was used to capture the LII signal precisely. The laser fluence was maintained at 0.4 J cm^−2^. The CMOS camera coupled with an intensifier was gated at 450 ns, and a delay of 760 ns after the occurrence of the laser pulse. Background subtraction was carried out to reduce background noise, and sheet image correction countered any inhomogeneity in the laser sheet.

The TSI Scanning Mobility Particle Sizer (SMPS) Electrostatic Classifier, Model 3082, was used for analysing the particle size distribution in the flame plume. This instrument scans for particle number concentration in a spectrum of size bins between 15 and 550 nm, using a Differential Mobility Analyzer (DMA) for the pre-set flow rates of sheath flow and aerosol flow. The measurement probe was kept at a height of 200 mm above the burner along the flame axis. The height was selected to safeguard the SMPS probe from the high-temperature closer to the flame. The sampling configuration adapted in this study may introduce coagulation, condensation, diffusional wall losses, and thermophoretic effects. Which in turn may influence the measured particle diameters and total number concentrations. However, since all measurements were conducted using an identical sampling configuration, the reported SMPS data represent the sampled aerosol properties and are interpreted qualitatively in terms of relative changes.

Furthermore, the SMPS size distribution measurements for all experiments were made under the same ambient sampling conditions, without the aid of external dilution, thermodenuder, or catalytic stripper. In all cases, the particle number concentrations were sufficiently below the SMPS saturation limit, without separating particles based on volatility. Without volatility-based removal, the size distributions reported may be affected by semi-volatile aerosol species. However, the effects of volatile species on the nanoparticle size distribution are expected to be uniform across all cases. Thereby revealing the effect of parameter variation through comparison between SMPS data without measuring the absolute particle numbers.

## 3. Results and discussion

### 3.1. Strand burner characterization

An initial set of experiments was performed to characterize the strand burner. If a compensating fuel supply is not provided, the fuel level decreases continuously during the experiment. Correspondingly, after an initial rapid transient increase, the visible flame height continuously reduces until extinction. The variation in flame height was attributed to the variation in the evaporation rate of fuel in the strand, which is inversely correlated with the depth of the fuel in the tube.

**Fig. 2:**
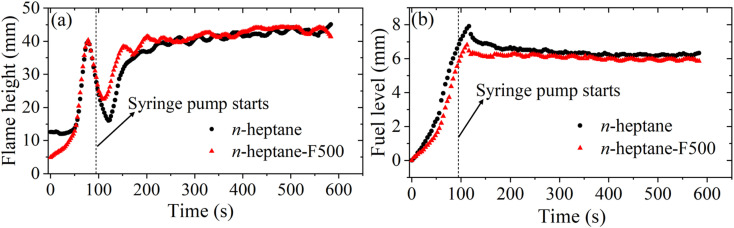
Variation of (a) flame height and (b) fuel level.

When using optical diagnostics such as LII and extinction-based line-of-sight calibration, a dynamic flame structure or large fluctuations in the flame can impair the calibration and measurement accuracy. In order to obtain a relatively stable flame, a constant supply of the liquid fuel to the strand was provided using a syringe pump. In the current study, a pumping rate of 0.22 mL min^−1^ was chosen. At this pumping rate, the flame height was found to approach the maximum height that could be investigated using our LII system. The effective flame height during the experiments was approximately 42 mm for pure *n*-heptane as well as *n*-heptane-F500. The corresponding fuel level in the strand was approximately 6 mm below the tip of the tube. Fig. 2 shows the steady flame height and fuel level obtained with the constant pumping of the fuel.

Although the flame under these conditions was sufficiently stable for diagnostic purposes, the flame still showed a fluctuating behavior, most clearly visible at the tip. This fluctuation may be a combination of buoyancy-driven instabilities, disturbances in local temperature and flow fields, and pulsations from the pumping action of the syringe pump. The typical standard deviation in the flame height was approximately 1 mm or about 2.5% of the average flame height. The relatively small magnitude of the fluctuation, availability of excess laser sheet thickness, localization of the fluctuations at the tip of the flame, and averaging of LII measurements over sufficiently long durations would minimize the impact of these fluctuations on the results reported.

### 3.2. Ferrocene and *n*-heptane co-evaporation

The iron release mechanism observed in ferrocene doped diffusion flames involves the thermal decomposition of the additive within the high-temperature zone of the flame. In the current study, the additives are dissolved in liquid *n*-heptane which burns *via* a strand burner setup similar to a liquid pool fire. Hence, before thermal decomposition, the vaporization characteristics of the dissolved additives may determine the gas phase dosing despite identical liquid phase concentration. However, in the current study, uniformity in liquid-phase concentration may be a reasonable representation of gas-phase dosing.

Under our experimental conditions, fuel as well as additive transport to the flame zone is governed by phase change equilibrium mechanics. Here, the maximum liquid phase temperature may be approximated to be 98.4 °C which is the boiling point of *n*-heptane at atmospheric pressure.^26^ All three additives are thermally stable at temperatures much higher than this value^27–29^ and can be expected to remain fully soluble in the base fuel given their high solubility in non-polar hydrocarbon solvents.^30,31^ Thus, the possibility of thermal decomposition before flame zone or precipitation into liquid phase due to solubility changes are unlikely. Also, ferrocene is known to possess significant vapor pressure to allow co-evaporation with hydrocarbon solvents near their boiling point.^32,33^

**Fig. 3:**
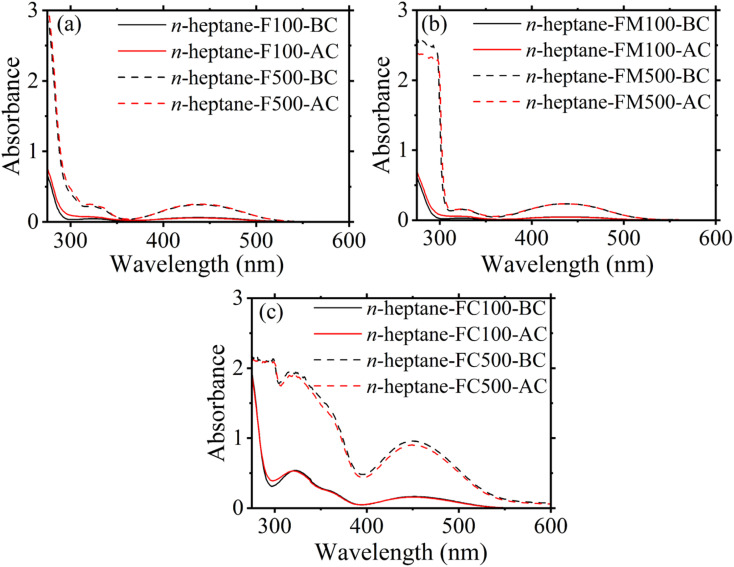
UV-Vis spectroscopy data obtained for *n*-heptane doped with (a) ferrocene, (b) ferrocene methanol, (c) ferrocene carboxaldehyde.

In order to gauge possible changes in the composition of the liquid fuel within the strand as it undergoes the combustion process, UV-Vis spectroscopy was carried out for the fuel samples under study before (BC) and after (AC) combustion. Fig. 3(a)–(c) show the UV-Vis spectra for all compositions.

Each spectrum in Fig. 3 shows the two prominent absorbance peaks representing the presence of the organometallic additives in *n*-heptane. The low energy peak occurs between the wavelengths of 400 nm to 500 nm, corresponds to the d–d transition of the iron atom in the ferrocene molecule.^34^ The high-energy peak occurs between 300 to 400 nm and originates from the cyclopentadienyl rings of ferrocene.^35,36^ No peak shift was observed for any compositions before and after combustion, indicating their compositional stability inside the strand. These results suggest solvent co-evaporation and subsequent simultaneous participation in the combustion reaction. Furthermore, these findings support the assumption that uniformity in liquid-phase concentration may be a reasonable representation of gas-phase dosing.

Additionally, the difference in the volatility may be gauged by comparing the enthalpy of sublimation for ferrocene and ferrocene derivatives. The enthalpy of sublimation for ferrocene and ferrocene carboxaldehyde is reported as 73.1 kJ mol^−1^ and 89.9 kJ mol^−1^,^35^ respectively. While the value for ferrocene methanol is reported as 102.8 kJ mol^−1^.^37^ Since, under similar conditions and for similar substances, lower enthalpy of sublimation indicates higher concentration in gas phase, the additives in the current study will be ranked by increasing tendency for creating gas phase species as ferrocene > ferrocene carboxaldehyde > ferrocene methanol. However, the observed soot suppression trends do not follow the ranking based on volatility, suggesting that functional-group chemistry contributes to the soot suppression.

### 3.3. Soot concentration measurements

**Fig. 4:**
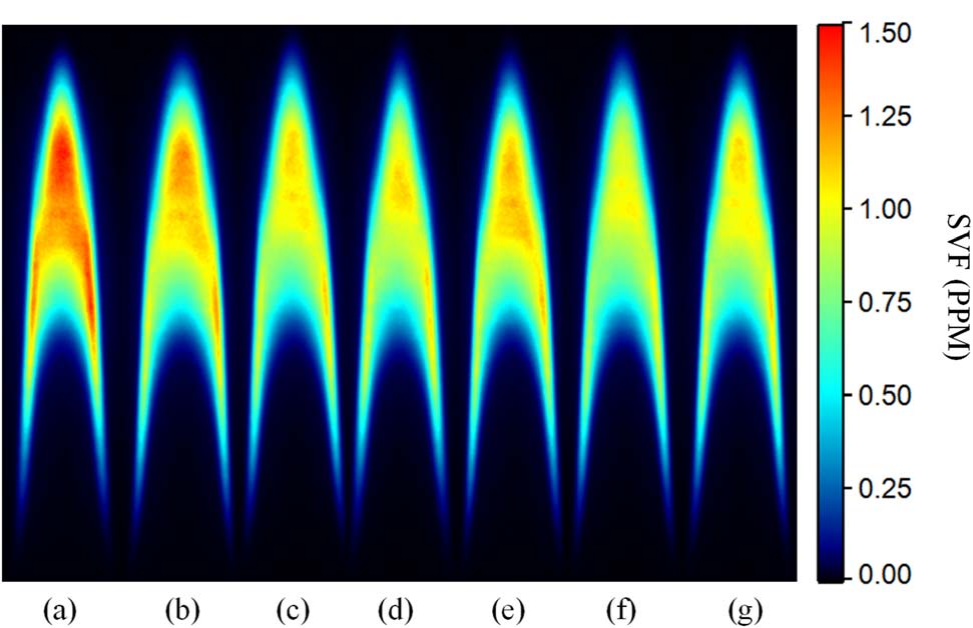
SVF contour comparison for (a) *n*-heptane (b) *n*-heptane-F100 (c) *n*-heptane-FM100 (d) *n*-heptane-FC100 (e) *n*-heptane-F500 (f) *n*-heptane-FM500 (g) *n*-heptane-FC500.

Fig. 4 shows the soot volume fraction (SVF) contours for the various fuel compositions. The measurements showed that the *n*-heptane flame has the highest SVF compared to the mixture with organometallic additives. As seen in Fig. 4, all additives reduce the SVF within the flame compared to the baseline of pure *n*-heptane with varying effectiveness. Furthermore, only a slight reduction in the SVF was observed as the concentration of the organometallic additive increased from 100 ppm to 500 ppm.

**Fig. 5:**
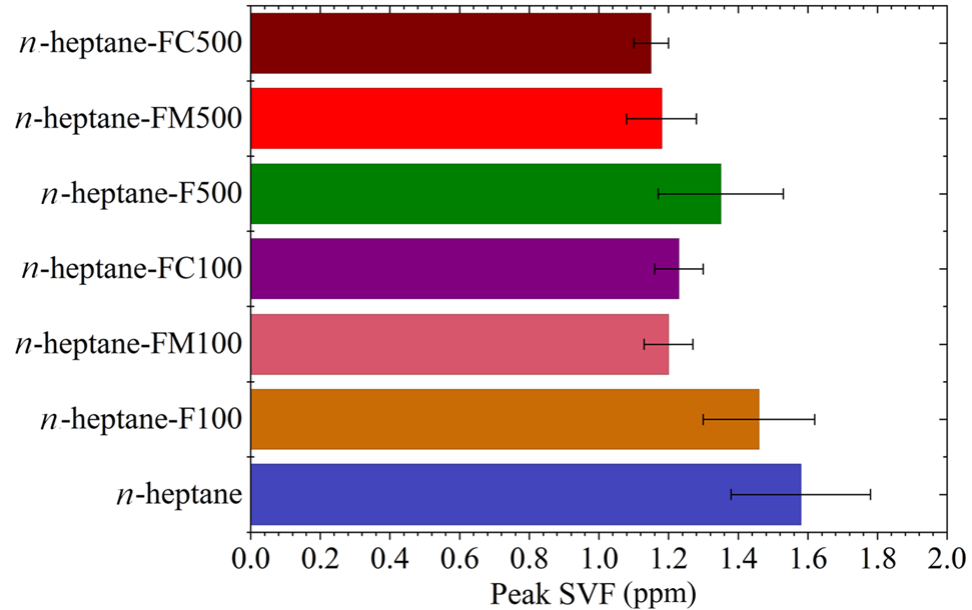
Variation of peak SVF for various samples.

The peak SVF in each case is shown in Fig. 5. The oxygenated ferrocene derivatives exhibited a higher soot suppression efficacy than ferrocene. At a 100 ppm concentration, ferrocene lowered the SVF by 8%, while ferrocene methanol and carboxaldehyde reduced the SVF by 24% and 22%, respectively. A concentration increase to 500 ppm improved the soot suppression by ferrocene to a 15% reduction, but provided only minor additional gains for the oxygenated derivatives.

**Fig. 6:**
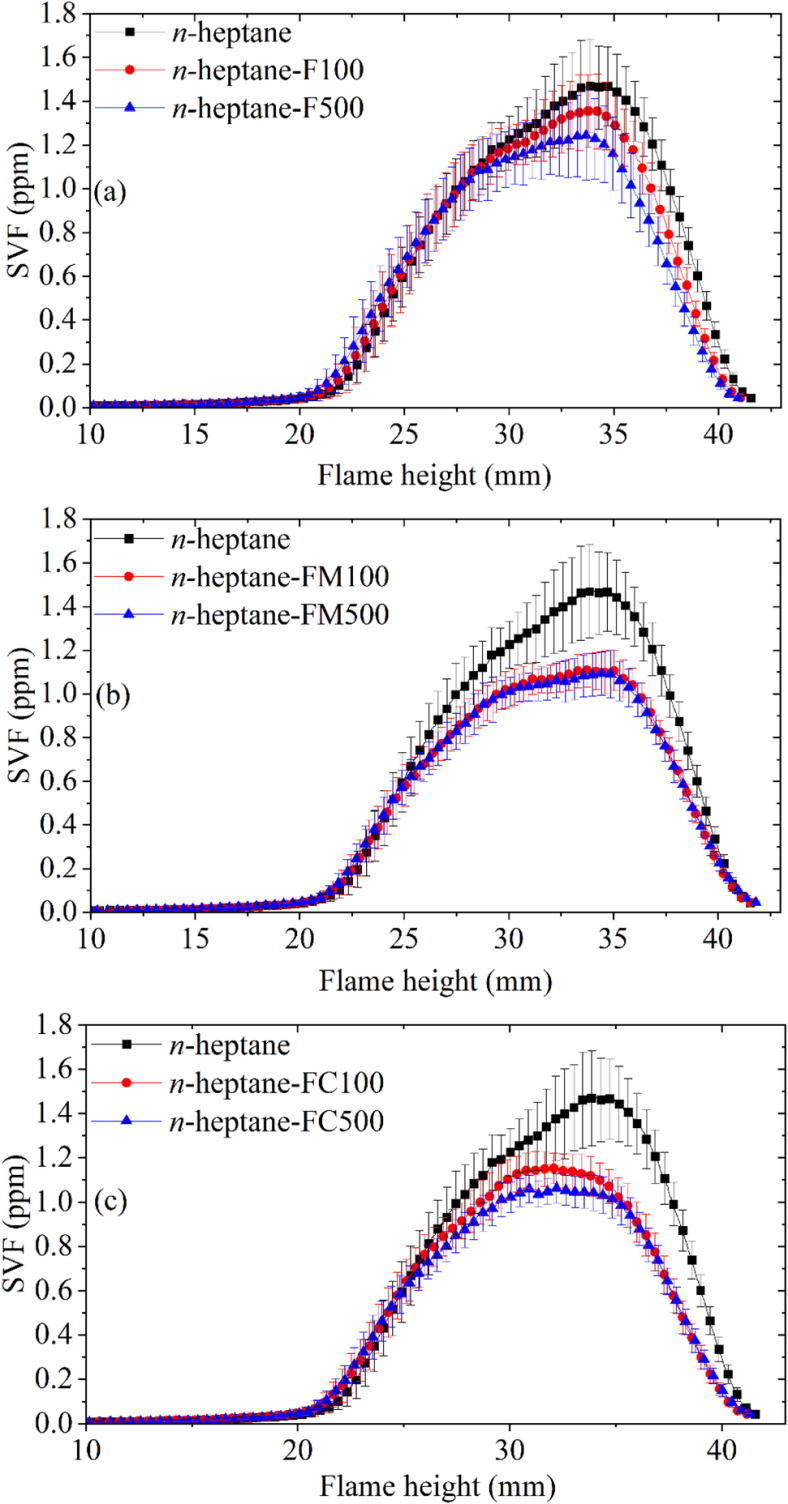
Axial SVF variation for (a) ferrocene (b) ferrocene methanol (c) ferrocene carboxaldehyde doped *n*-heptane compared with *n*-heptane.

The axial soot concentration profile was also investigated to reveal the soot inception, growth, and oxidation characteristics within the flame. The detailed axial SVF profile comparison is shown in Fig. 6. All profiles in Fig. 6(a)–(c), display the typical diffusion flame trend. In the case of a baseline pure *n*-heptane profile, careful observation reveals a gradually increasing trend in SVF beginning from 15 mm HAB, indicating the beginning of the soot inception zone. The sharp rise in SVF from approximately 20 mm HAB shows the soot growth zone. The soot concentration can be seen to plateau and then go through a sharp decline. The profile can be seen to reach a peak at approximately 35 mm HAB. A decreasing SVF profile beyond this point indicates the soot oxidation zone within the flame. The axial soot profiles for additive-doped flames show some deviation from the baseline profile of pure *n*-heptane. In the soot growth zone additive-doped flames show a slightly higher soot concentration between 20 and 25 mm HAB. Furthermore, the peak soot concentration for additive-doped flames is lower than the baseline case. In the case of ferrocene carboxaldehyde, the peak also appears to occur earlier, at approximately 32 mm HAB, when compared to *n*-heptane.

The possibility of LII calibration and SVF measurements being affected by the presence of iron-oxide nanoparticles was also examined. Although potential optical cross-sensitivity to the presence of metal-containing nanoparticles exists, it may be considered to be a minor phenomenon. Previous experimental studies^5,7^ have confirmed that iron oxide-based particles are present as inclusions within carbonaceous soot particles inside the flame zone, while standalone iron oxide particles may be detected only downstream of the flame. Therefore, in the present study, the calibration laser and the laser sheet used for SVF measurements predominantly encounter soot particles containing iron inclusions, without a significant independent population of iron nanoparticles within the measurement region.

Although modification of soot optical properties due to iron inclusion is possible, the fraction of iron contributing to iron-oxide nanoparticles is lower by several orders of magnitude than the carbon contributing to soot at the additive concentration levels used in this study. In typical laminar non-premixed hydrocarbon flames, only 1–5% of the fuel carbon is converted to soot for C_7_–C_12_ hydrocarbons under atmospheric conditions.^38,39^ Assuming 1% carbon conversion to soot and complete conversion of iron to iron-oxide, for the present heptane flow rate of 0.22 mL min^−1^ and additive concentration of 100 ppm, the resulting mass and volume fraction of iron oxide relative to soot remain negligibly small.

Furthermore, the absorption coefficient of soot at 532 nm is higher than that of iron and iron oxides.^40^ Therefore, the extinction of the calibration laser and the LII response are expected to be dominated by soot characteristics under the present conditions. Additionally, the LII detection timing in the current study was optimized for soot incandescence. Metal nanoparticles, owing to their higher thermal conductivity and smaller characteristic sizes, exhibit substantially faster cooling rates and reduced LII response compared to soot.^41,42^ Consequently, their contribution to the detected LII signal is expected to be minimal. Therefore, while some degree of cross-sensitivity may be present, available literature and scaling considerations indicate that the measurement from the LII experiments primarily reflects soot volume fraction.

### 3.4. Flame temperature measurement

**Fig. 7:**
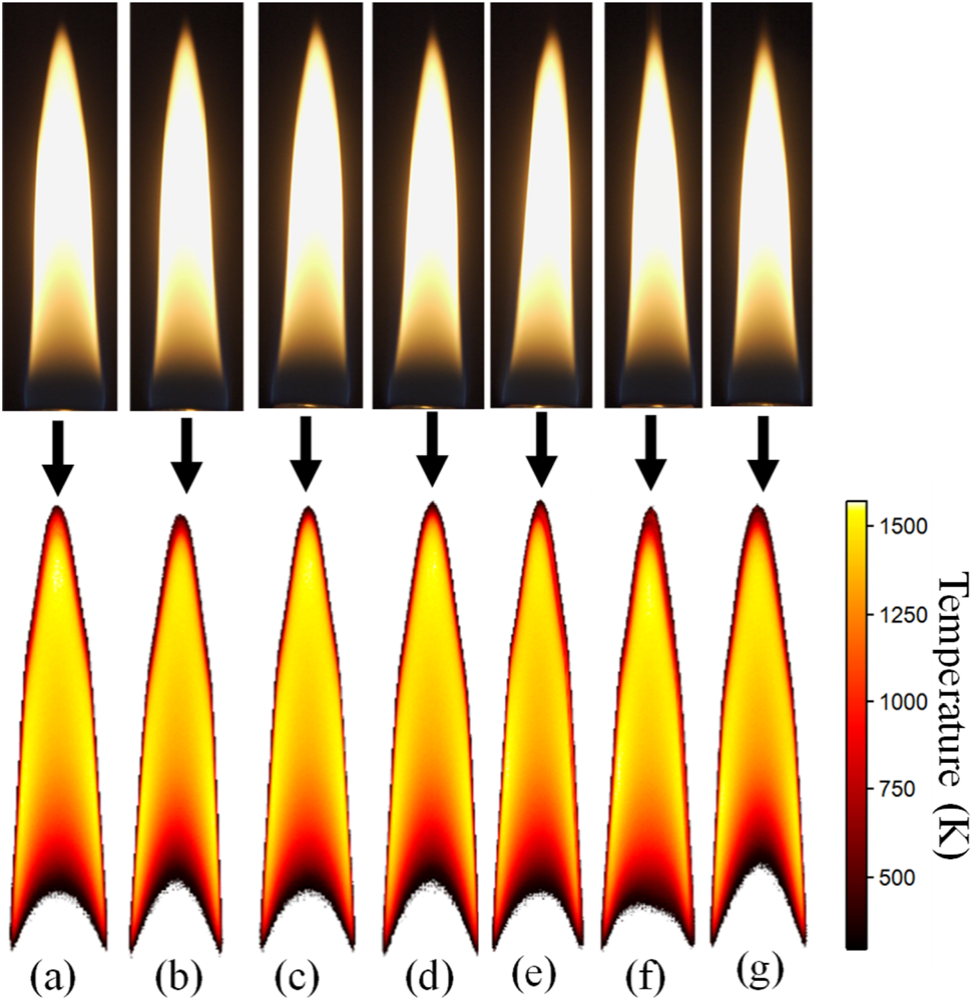
Flame temperature contours for (a) *n*-heptane (b) *n*-heptane-F100 (c) *n*-heptane-FM100 (d) *n*-heptane-FC100 (e) *n*-heptane-F500 (f) *n*-heptane-FM500 (g) *n*-heptane-FC500.

The contours for flame temperature averaged over 1 s are shown in Fig. 7 along with the actual flame images for various compositions. These temperature contours represent the instantaneous temperature field of the flame. The temperature contours were observed to be similar irrespective of the fuel composition. However, upon closer inspection and data analysis, differences in characteristics of the flame due to additives was revealed.

**Fig. 8:**
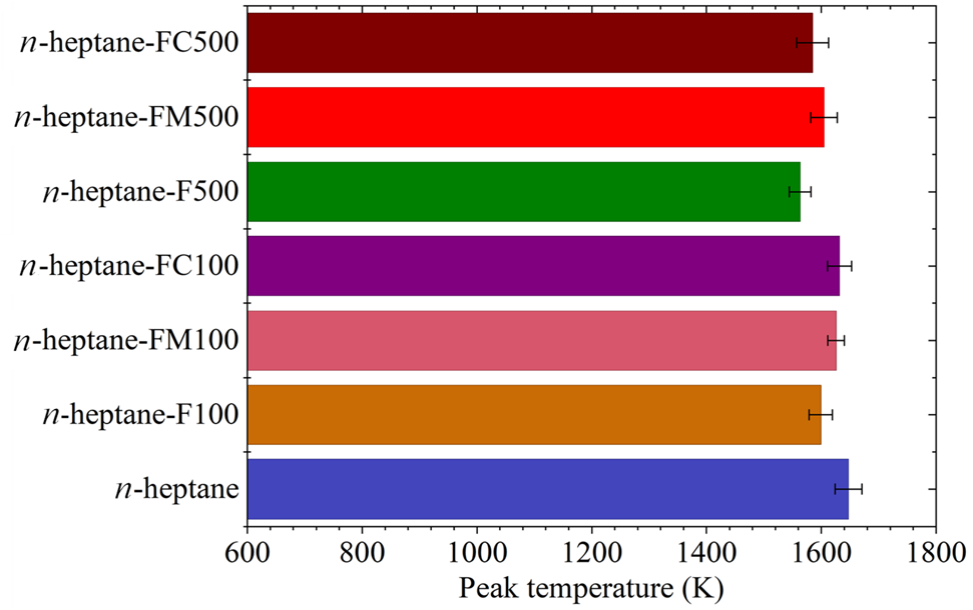
Maximum axial flame temperature for various flames.

Fig. 8 shows the variation of the maximum temperature along the flame axis for different fuel-additive mixtures. For pure *n*-heptane, the maximum temperature was 1647.6 ± 23.2 K. With the addition of 100 ppm additives, the temperature dropped by approximately 48 K for ferrocene, 22 K for ferrocene methanol, and 16 K for ferrocene carboxaldehyde. At the additive concentration of 500 ppm, the temperature reduced by 84 K for ferrocene, 43 K for ferrocene methanol, and 63 K for ferrocene carboxaldehyde.

### 3.5. Emission characterization

**Fig. 9:**
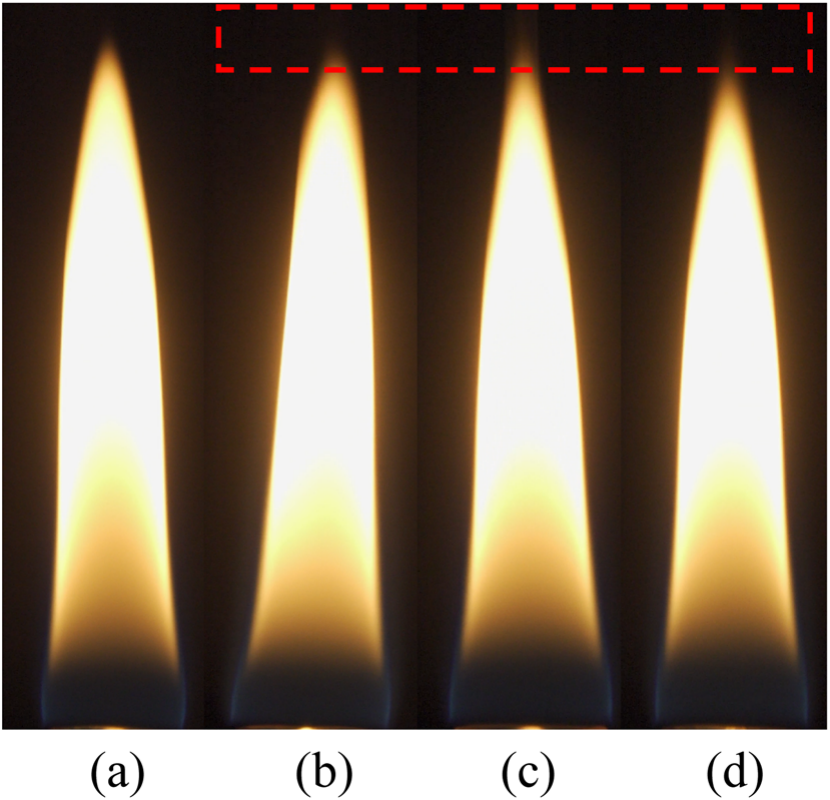
Flame images of (a) *n*-heptane (b) *n*-heptane-F500 (c) *n*-heptane-FM500 (d) *n*-heptane-FC500.

Although the experiments were conducted below the smoke point for *n*-heptane, the flames of 500 ppm additive concentration showed visible emissions as seen in Fig. 9.

**Fig. 10:**
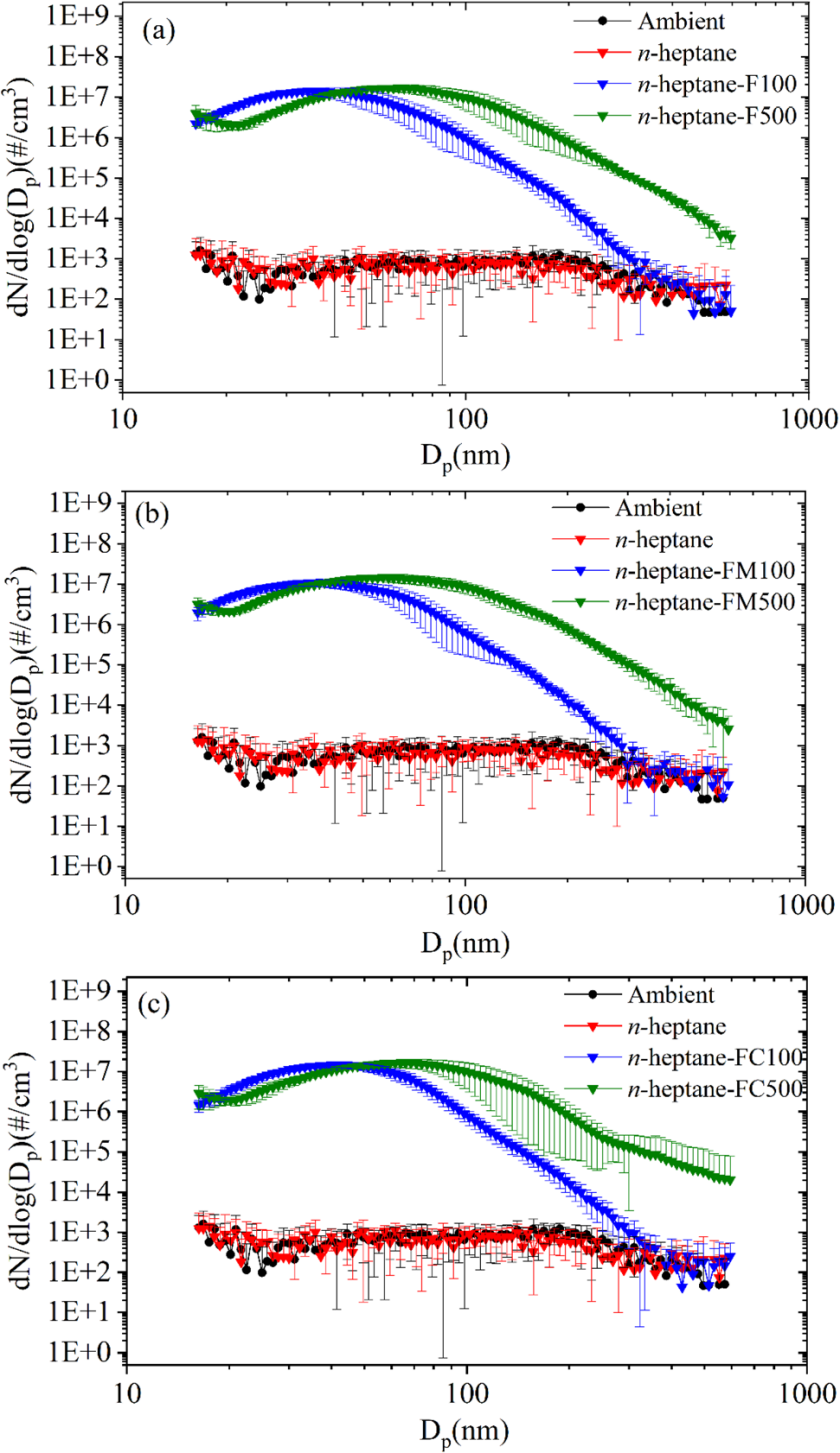
Particle number concentration *vs.* size comparison for (a) ferrocene (b) ferrocene methanol (c) ferrocene carboxaldehyde, doped *n*-heptane compared with *n*-heptane.

Fig. 10 shows the emission data obtained with SMPS with ambient baseline. The data indicated the presence of particulate emissions for additive-doped flames. The *n*-heptane flames showed no additional emissions against the baseline since the *n*-heptane combustion occurred below the smoke point. Compositions with 100 ppm additive concentration showed an emission peak around 35 nm in the particle mobility diameter. The compositions with 500 ppm concentration showed a shift in the peak to 65 nm as shown in Fig. 10(a)–(c).

However, since no ultrafine emissions were observed in the baseline *n*-heptane flame under identical experimental conditions, the emergence of a distinct ultrafine particle emission mode for ferrocene doped samples was attributed to iron-based nanoparticles originating from ferrocene. The attribution to is strongly supported by previous studies^5,7^ that have reported measurements of iron and iron-oxide nanoparticles formed in ferrocene-doped hydrocarbon flames.

**Fig. 11:**
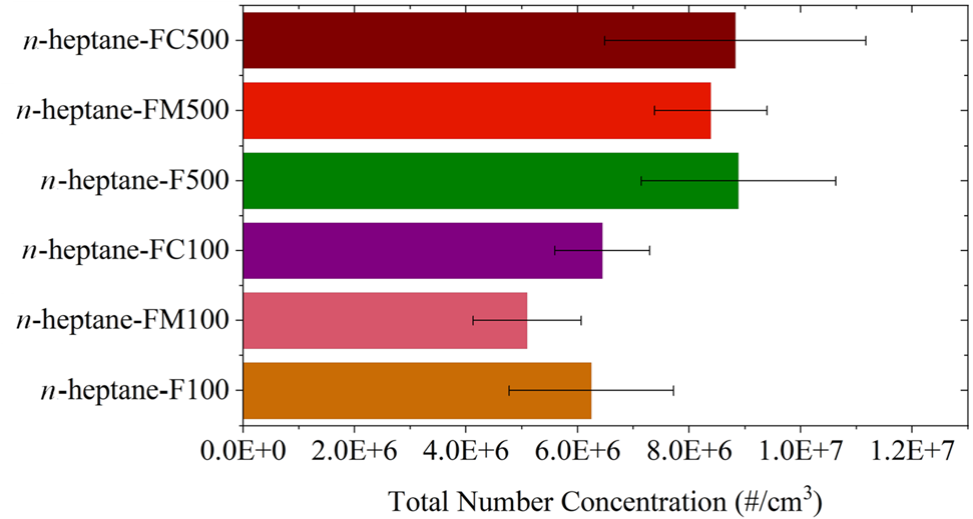
Total number concentration for the different additives.

Furthermore, Fig. 11 shows the calculated Total Number Concentration (TNC) for particulate emissions. Amongst the various additives, ferrocene and ferrocene carboxaldehyde display comparable TNC, while ferrocene methanol shows a lower TNC. The TNC can also be seen to increase with the concentration of the additive. It should be noted that while absolute values may be subject to sampling bias due to the limitations of the experimental technique, the relative differences and the emergence of an additional ultrafine emissions upon ferrocene addition remains attributable to changes in flame chemistry rather than sampling error.

## 4. Analysis

The combined results acquired from LII, pyrometry, and SMPS experiments elucidate the characteristic behaviour of the ferrocene-doped flames. The SVF contours and axial profiles establish that pure *n*-heptane produces the highest overall flame SVF among the tested fuel compositions. The introduction of ferrocene-based additives resulted in two distinct deviations from the pure *n*-heptane baseline in the soot growth zone and in the soot oxidation zone as seen in Fig. 6. In the soot growth zone, all additive-doped flames displayed a marginally higher soot concentration. Subsequently, the SVF profiles for ferrocene-added samples peaked earlier and at a significantly lower SVF value compared to pure *n*-heptane, followed by a rapid decay. The results clearly demonstrate the increased soot suppression affected by the oxygenated ferrocene derivatives. The SMPS experiments invariably showed an increase in the total number concentration ultrafine particle emissions with increasing additive concentration. Lowest amount of nanoparticle emissions was observed for ferrocene methanol, while the TNC of particulate emissions for ferrocene and ferrocene carboxaldehyde were comparable.

The flame temperature measurements indicated that all additive-doped flames exhibit a reduced axial peak temperature relative to the baseline of pure *n*-heptane. The axial peak temperature, which occurs just before the flame terminus, indicates the temperature related to the soot oxidation zone. The juxtaposition of the trends of peak axial SVF given in Fig. 5 and peak axial temperatures given in Fig. 8 highlights an interesting trend. The data shows, ferrocene and ferrocene carboxaldehyde cause a drop in temperature and corresponding reduction in peak SVF both increasing with additive concentration. However, ferrocene methanol shows negligible variation in these metrics despite increasing the concentration.

The in-flame soot suppression, emission of ultrafine particles, and reduction in flame temperature are features common to all cases studied here.

The most pronounced effect is observed in the soot oxidation zone near the flame tip, where a reduction in peak SVF is measured for all samples. This phenomenon is consistent with the enhancement of soot oxidation due to catalysis by the iron-oxide species.^11,12^ The fuel samples with oxygen-containing functional groups in the ferrocene structure showed an enhanced soot suppression as shown in Fig. 6. This effect suggests that oxygenated functional groups may further promote oxidation, potentially by modifying local reaction mechanism. The literature consistently reports oxygenated functional groups leading to reduced soot formation in diffusion flames by suppressing aromatic precursor growth and enhancing oxidation reactivity.^15–17^ Along the same lines, the higher SVF in the soot growth zone is also consistent with modified precursor chemistry, altered radical concentrations, and effects due to iron-based particles. Given the appearance of nanoparticle emissions after ferrocene addition, they could be attributed to formation of iron-based nanoparticles. Studies directly detecting the iron-based particles in the flame^5,7^ as well as studies indicating presence of iron within the carbonaceous matrix of the soot^4,8,9^ have given ample evidence of this action. Although direct measurements confirming the composition will be part a future study, the systematic trends observed across additives and robust studies reported in the literature, indicate that iron-derived species lead to both soot suppression and ultrafine particle emissions.

The reduction in flame temperature in the soot oxidation zone could indicate accelerated soot oxidation, altered radiative heat transfer, modified flame structure, or transport effects. However, given the low additive concentration and unchanged bulk burning rate, large-scale flame restructuring is unlikely. Furthermore, the well-established catalytic action of the iron-based particles and reactive species from oxygenated ferrocene are known lower the activation energy required for soot oxidation, allowing the oxidation to occur at a lower temperature.

The increasing trend in soot suppression and flame temperature reduction for ferrocene and ferrocene carboxaldehyde indicate a primarily iron-based catalytic action as the driving force. While the insensitivity of ferrocene methanol to increasing additive concentration may indicate a saturation of iron-catalysis pathway or a modification of the iron-species by OH group. Ferrocene methanol can be expected to release oxygenated fragments, which strongly promote oxidation of soot, leading to a lower SVF compared to pure ferrocene but higher flame temperature due to increased availability of oxygen. In the case of ferrocene carboxaldehyde, the decomposition of the additives may lead to fragments containing carbon and fewer oxygen radicals than ferrocene methanol. Thus, ferrocene carboxaldehyde undergoes a subdued radical-driven oxidation, producing lower reduction in SVF than ferrocene methanol. Functionalization effects further suggest that the OH-containing derivative mitigates ultrafine particle emissions relative to non-functionalized ferrocene, possibly through altered iron oxide chemistry.

The combined evidence indicates that ferrocene-based additives suppress soot within the flame through catalytic oxidation, with oxygenated functional groups enhancing suppression. The additives also introduce nanoparticle emissions which could be attributed to iron-containing species. Although contributions from alternate mechanism, altered flame structure, or transport effects cannot be fully excluded without additional diagnostics such as direct measurements of radical species or PAHs, and nanoparticle composition, the observed phenomena are consistent with catalytic oxidation of soot particles by iron-containing species and action of oxygenated functional groups. This study focuses on experimentally resolved trends in soot evolution induced by organometallic addition to *n*-heptane diffusion flame, rather than on isolating individual chemical pathways, which will be the subject of future work.

## 5. Conclusion

This study experimentally demonstrated that ferrocene-based organometallic additives are effective in suppressing soot in *n*-heptane diffusion flames. The soot suppression was attributed to enhanced soot oxidation at the flame tip due to the inclusion of iron-based nanoparticles and oxygenated reactive species. Pure *n*-heptane produced the highest SVF, whereas the additives, particularly ferrocene methanol and ferrocene carboxaldehyde, reduced SVF by up to 24%. While increasing the additive concentration from 100 ppm to 500 ppm offered only marginal gains in SVF reduction, the total particle number concentration significantly increased due to nanoparticle emissions observed after ferrocene addition. These ultrafine emissions are attributed to iron-containing nanoparticles based on strong evidence reported in the literature. This finding indicates a trade-off between in-flame soot suppression and the increased emission of ultrafine particulate matter. Future research will focus on positively identifying the composition of the nanoparticles, and optimizing additive structures to minimize nanoparticle formation while preserving the substantial soot inhibition benefits.

## Conflicts of interest

There are no conflicts to declare.

## Supplementary Material

RA-016-D5RA09720G-s001

## Data Availability

All data generated or analyzed during this study are included in this article and its supplementary information (SI). No additional datasets were created or used beyond those presented here. Supplementary information: raw data for temperature measurement, raw data for soot concentration measurement, raw data of UV-Vis spectra. See DOI: https://doi.org/10.1039/d5ra09720g.
